# Polybenzoxazine/Polyhedral Oligomeric Silsesquioxane (POSS) Nanocomposites

**DOI:** 10.3390/polym8060225

**Published:** 2016-06-07

**Authors:** Mohamed Gamal Mohamed, Shiao-Wei Kuo

**Affiliations:** Department of Materials and Optoelectronic Science, National Sun Yat-Sen University, Kaohsiung 80424, Taiwan; mgamal.eldin12@yahoo.com

**Keywords:** polybenzoxazine, POSS, nanocomposites, hydrogen bonding

## Abstract

The organic/inorganic hybrid materials from polyhedral oligomeric silsesquioxane (POSS, inorganic nanoparticles) and polybenzoxazine (PBZ) have received much interesting recently due to their excellent thermal and mechanical properties, flame retardance, low dielectric constant, well-defined inorganic framework at nanosized scale level, and higher performance relative to those of non-hybrid PBZs. This review describes the synthesis, dielectric constants, and thermal, rheological, and mechanical properties of covalently bonded mono- and multifunctionalized benzoxazine POSS hybrids, other functionalized benzoxazine POSS derivatives, and non-covalently (hydrogen) bonded benzoxazine POSS composites.

## 1. Introduction

**Figure 1:**
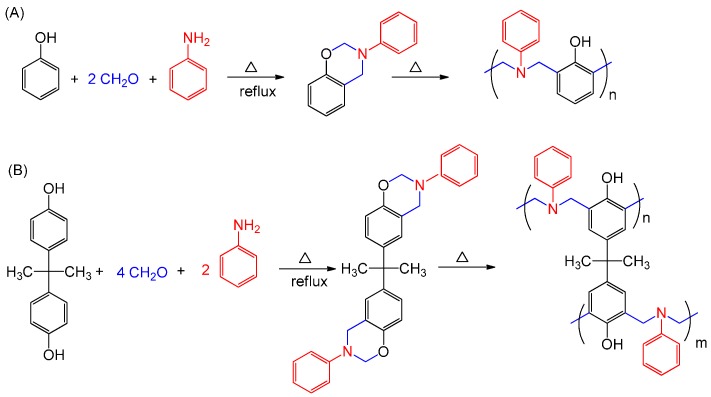
Preparation and thermally induced ring-opening polymerization of (**A**) P-a and (**B**) B-a types of BZ monomers.

Benzoxazine (BZ) monomers are heterocyclic compounds containing oxygen and nitrogen atoms in a six-membered oxazine ring; they can be synthesized through facile and environmentally friendly condensation reactions of aromatic phenols, primary or aromatic amines, and paraformaldehyde in the absence of a catalyst [1,2,3,4,5,6,7,8,9,10,11,12,13,14,15,16,17,18,19,20]. Polybenzoxazines (PBZs) could be obtained through thermally activated ring-opening polymerizations from BZ monomers, as displayed in Figure 1 [21,22,23,24,25,26]. PBZs are phenolic resin–like materials and thermosetting polymers that possess attractive dielectric constants (*k*), excellent thermal, chemical, and mechanical properties, flame-retardance, low moisture absorption, good heat resistance, and flexibility in molecular structural design [27,28,29,30,31,32,33,34]. In addition, PBZs comprise a new class of non-silicon or non-fluorine polymer materials with low-surface-free-energy properties; they have wide applications as mold release materials in nanoimprint technology, in lithographic patterning, and as a superhydrophobic surface material due to their strong intramolecular hydrogen bonding after thermal curing polymerization [31,35,36,37,38,39,40]. In some cases, PBZs have properties superior to those of some traditional thermosets (including epoxies, bismaleimides). Nevertheless, PBZs can possess some unattractive characteristics-for example, the high temperature need for complete ring opening polymerization and the brittleness of the cured materials compared with those of other thermoset crosslinking materials, restricting their applications as matrices for some high-performance composites [41,42]. The incorporation of organic functional groups (e.g., vinyl, allyl, methacryloyl, nitrile, benzoxazole, epoxy), inorganic silicates (e.g., clay), carbon nanotubes (CNTs), and polyhedral oligomeric silsesquioxane (POSS) into BZ monomers can improve the thermal or mechanical properties of PBZ resins through the crosslinking network formation and decreased chain mobility [43,44,45,46,47,48,49,50,51,52,53,54,55,56].

Both organic components and inorganic POSS can display the enhancement of performance capability compared with that of their non-hybrid polymeric materials [57,58]. The molecular structure of POSS features a silica cage as the core (a Si–O–Si framework of nanoscale size) with the other organic functional group covalently bonded at the cage corners (*i.e*., each Si atom); thus, it consists of the inorganic siloxane group as the inner core and the polar or nonpolar organic groups as an outer layer. POSS nanostructures typically have the empirical chemical formula RSiO_1.5_, where R could be the hydrogen or the organic moiety such as alkyl, alkylene, arylene, aryl, acrylate, epoxy or hydroxyl units [59,60,61,62]. They have attracted much recent attention because of their nanometer-scale dimensions (diameter: 1–3 nm), their organic/inorganic hybrid structures, and excellent mechanical and thermal properties [63,64,65,66].

**Figure 2:**
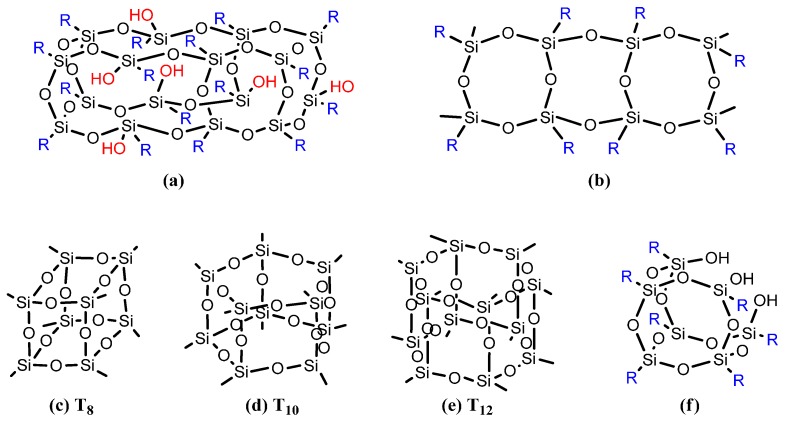
Chemical structures of silsesquioxanes: (**a**) random structure, (**b**) ladder structure, (**c**) T_8_, (**d**) T_10_, (**e**) T_12_ cage structure, and (**f**) partial cage structure [59]. Reproduced with permission from Elsevier.

Scott was the first to synthesize oligomeric organosilsesquioxanes (CH_3_SiO_1.5_)*_n_* through thermolysis of the polymer materials afforded by the co-hydrolysis of methyltrichlorosilane and dimethylchlorosilane [67]. POSS structures can be divided into monofunctional (when only one of the organic groups is reactive) and multifunctional (when more than one organic group is reactive) derivatives [68,69,70]. Silsesquioxane nanostructures include random, cage, partial cage, and ladder structures (Figure 2) [59,70,71,72]. In general, monofunctional POSS derivatives (R´R_7_Si_8_O_12_) are synthesized through (i) co-hydrolysis of trifunctional organo- or hydrosilanes [73,74], (ii) corner-capping reactions [75], and (iii) substitution reaction with the siloxane cage retention [76,77]. On the other hand, multifunctional POSS derivatives are typically prepared via the Pt-catalyzed hydrosilylation with (HSiO_1.5_)_8_ or octakis(dimethylsiloxy)silsesquioxane [(HMe_2_SiOSiO_1.5_)_8_, Q_8_M_8_^H^] cage with alkenes or alkynes [78,79,80,81]. The dispersion and incorporation of POSS nanoparticles (NPs) within polymers to form organic/inorganic hybrid materials without surface treatment is a particularly active field of research in academia and industry because these NPs have zero dimensionality (1-, 2-, or 3-D scaffolds), well-defined structures, high temperature-stability, monodisperse molecular weights, ultra-low dielectric constants, and greater design flexibility relative to conventional fillers (for example, clay, graphene, carbon nanotube and boron nitride) [81]; in addition to the sizable interfacial interaction between the polymer segments and composite particles, these hybrids have several applications in polymer electrolytes, drug delivery, and as thermosetting polymers [82,83,84,85,86,87,88,89,90]. The dispersion of POSS NPs into a polymer matrix can enhance its rigidity, modulus, and strength, while decreasing its flammability and viscosity [36,88,89]. Two approaches are generally used to incorporate the POSS NPs into polymer materials: (i) chemical crosslinking or chemical copolymerization, where the POSS nanostructures are attached to the polymer through covalent bonds [91], and (ii) physical blending, where POSS NPs are physically bonded with the polymer through solvent-casting or melt-mixing. Such physical blending approaches depend strongly on the compatibility and processability of the POSS with the polymer matrix; these phenomena can be enhanced through the connection of vertex groups to the silicon atoms [31,92]. Accordingly, many attempts have been made to control the locations of the NPs in POSS-containing polymer nanocomposites. The difference between the chemical crosslinking and physical blending approaches is the absence of macro-phase separation between the polymer matrix and the POSS NPs in the former, due to the linkage through covalent bonds [93].

The incorporation of POSS NPs into a polymer matrix can be performed through several organic reactions, including hydrosilylation, condensation, and grafting [59]. Many different types of POSS compounds have been synthesized, including octa(aminophenyl)silsesquioxane (OAPS) [92,94], incomplete-cage POSS [95,96], epoxide octavinyl-POSS (EOVS) [97], and octa(3-chloroammoniumpropyl)-POSS (OCAPS) [98]. These novel POSS materials can be used to prepare various POSS-containing PBZ nanocomposites through either chemical crosslinking or physical blending. The incorporation of POSS NPs into PBZ matrix allows the development of new classes of organic/inorganic hybrid nanocomposite materials potentially possessing unique properties, making the field one of the most interesting in materials science. In this review, we focus on the synthesis and characterization of various kinds of POSS-containing PBZs, including those derived covalently and noncovalently (such as, hydrogen bonded) from mono- and multifunctional POSS as well as through physical blending with PBZs.

## 2. Preparation of PBZs Containing POSS Nanocomposites

Three approaches have been developed for the introduction of POSS NPs into BZs monomers for the synthesis of PBZ-POSS: (i) using Q^8^H^8^, OAPS, HPOSS, and amine-POSS as precursors; (ii) incorporating POSS structures as crosslinkers to increase the crosslinking density of the PBZ resins [e.g., allowing the reactive groups of OAPS (amino group), EOVS (epoxy group), and trisilanol POSS (T7POSS) to react with BZ monomers during thermal curing polymerization]; and (iii) incorporating POSS derivatives as catalysts to accelerate the ring-opening polymerization of BZ monomers through the release of free acid or amino groups (for example, from octa(p-toluenesulfonic acid ammonium salt) polyhedral oligomeric silsesquioxane (OPAAS POSS)) at elevated temperature [99]. Yu *et al.* successfully synthesized the diverse array of POSS materials that they used to modify PBZ resin; these hybrid materials exhibited mechanical and thermal properties superior to those of pristine BZ monomers [100].

### 2.1. Monobenzoxazine-Functionalized POSS (BZ-POSS)

**Figure 3:**
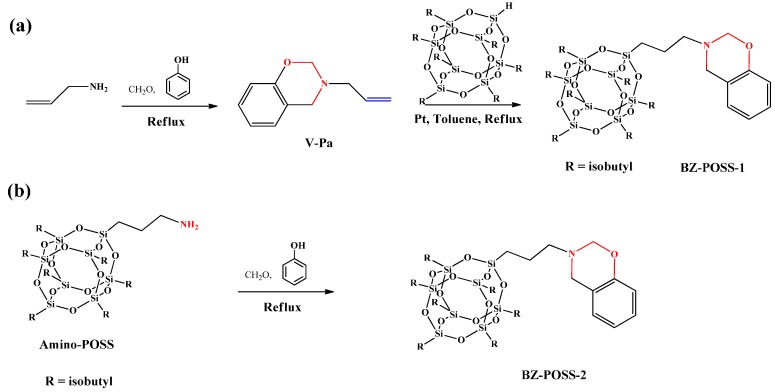
(**a**) The mechanism and the corresponding chemical structure for the synthesis of the VP-a monomer, as well as the preparation of the BZ-POSS-1 macromonomer through hydrosilylation; (**b**) synthesis of the BZ-POSS-2 macromonomer from amino-POSS [105]. Reproduced with permission from Elsevier.

**Figure 4:**
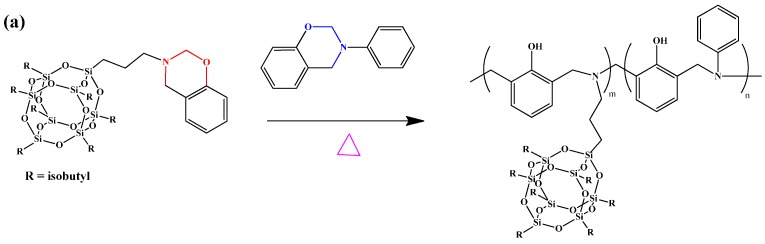

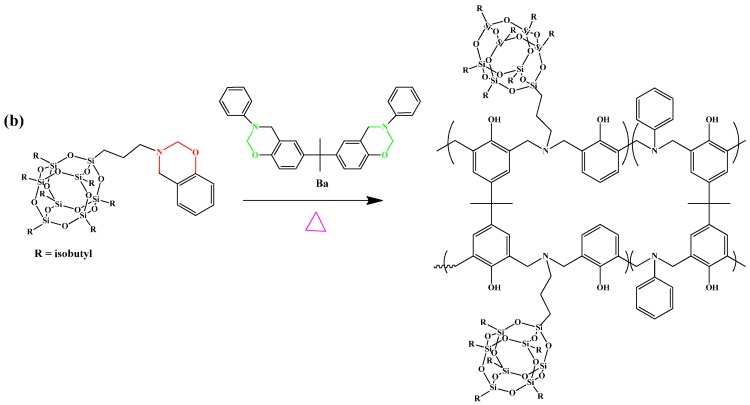
Preparation of PBZ/POSS nanocomposites from BZ-POSS monomers containing (**a**) P-a and (**b**) B-a type BZ monomers [105]. Reproduced with permission from Elsevier.

Monofunctional POSS derivative is the most useful compound for copolymerization with other monomers through free-radical polymerization, living anionic polymerization or controlled living radical polymerization such as atom transfer radical polymerization (ATRP) or reversible addition fragmentation chain transfer polymerization (RAFT) for the preparation of high-performance POSS-containing polymers [101,102,103,104]. We first synthesized mono-functionalized BZ ring containing POSS (BZ-POSS) using two approaches (Figure 3) [105]: (i) the preparation of vinyl-terminated BZ and then hydrosilylation with POSS to afford BZ-POSS-1 and (ii) condensation of a primary amine-containing POSS (amine-POSS) with formaldehyde and phenol in THF solution at 90 °C to obtain BZ-POSS-2. The chemical structure of BZ-POSS-2 was investigated using ^1^H NMR and FTIR spectroscopy and size exclusion chromatography. Subsequent copolymerization of BZ-POSS-1 with 3-phenyl-3,4-dihydro-2H-benzoxazine (Pa) and 6,6′-(propane-2,2-diyl)bis(3-phenyl-3,4-dihydro-2H-benzoxazine) (Ba) BZ monomers by thermally activated ring-opening polymerizations afforded Pa-POSS and Ba-POSS copolymers, respectively (Figure 4).

According to thermogravimetric analysis (TGA), increasing the BZ-POSS contents from 0 to 10 wt % caused the degradation temperature (*T*_d_) and char yield of the Ba-POSS and Pa-POSS copolymers to increase, due to the POSS nanostructures restricting the mobility of polymer chains at elevated temperature. The thermal stability of the POSS copolymers decreased upon adding more than 10 wt % of BZ-POSS because of the macrophase separation and gross aggregation of BZ-POSS that occurred prior to polymerization.

### 2.2. Multibenzoxazine-Functionalized POSS

**Figure 5:**
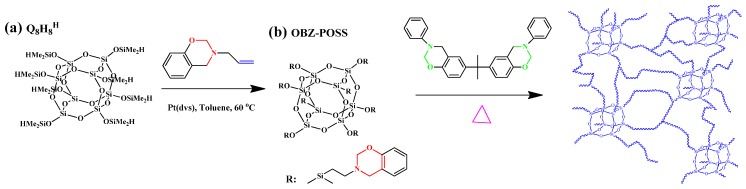
Preparation of multi-functionalized (OBZ POSS) (**a**) through hydrosilylation from Q_8_M_8_^H^ (**b**) with VB-a monomer and subsequent formation of PBZ/POSS nanocomposites with a network structure [107]. Reproduced with permission from Elsevier.

**Figure 6:**
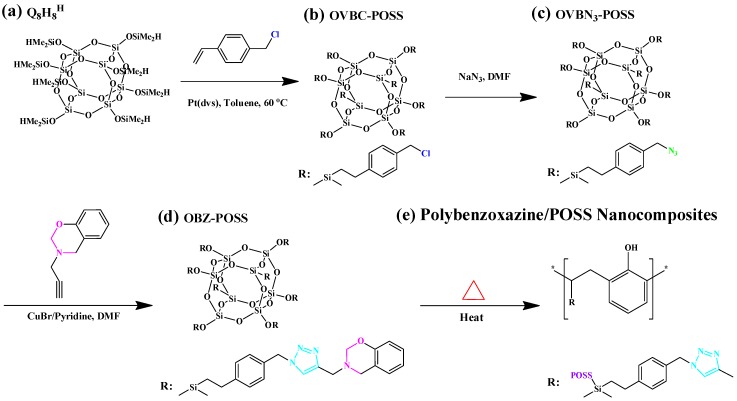
The hydrosilylation of vinyl benzyl chloride monomer with Q_8_M_8_^H^ (**a**) to form OVBC-POSS (**b**) and OVBN_3_-POSS (**c**) and the click reaction to give OBZ-POSS (**d**) and thermal curing to give PBZ/POSS nanocomposite (**e**) [108]. Reproduced with permission from Elsevier.

Multifunctional POSS/BZ monomers have been synthesized in high purity and yield by many research groups. We synthesized our first reported multifunctionalized POSS presenting eight organic BZ tether units (OBZ-POSS) via the hydrosilylation of a vinyl-terminated BZ monomer (VP-a) with Q_8_M_8_^H^ by using the platinum complex (Pt-dvs) as catalyst (Figure 5) [106,107]. The thermal stability in the resulting PBZ/POSS nanocomposites was evidenced by the increased decomposition temperatures of Pa-POSS and Ba-POSS nanocomposites upon increasing the OBZ-POSS contents [106]. We have also synthesized new multifunctional BZ-containing POSS (OBZ-POSS) through 1,3-dipolar cycloaddition between an octaazido-functionalized POSS (OVPN_3_-POSS) and 3,4-dihydro-3-(prop-2-ynyl)-2*H*-benzoxazine (P-pa) (Figure 6). The decomposition temperature (*T*_d_) and char yield of OBZ-POSS nanocomposites, determined through TGA analysis, were higher than those of P-pa and BA-m [108].

**Figure 7:**
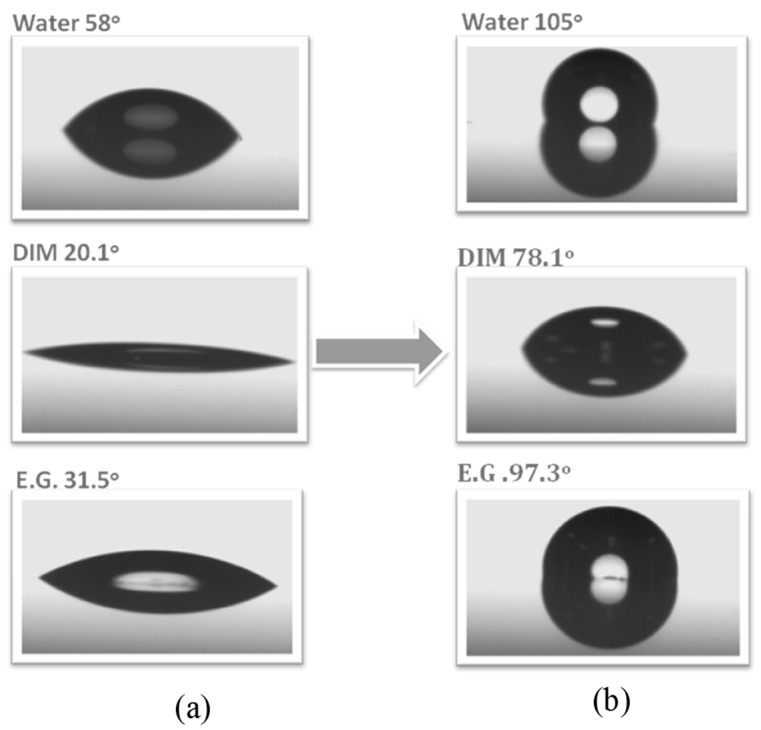
Advancing contact angles for water, diiodomethane (DIM), and ethylene glycol (EG) of (**a**) a P4VP thin film and (**b**) surface modified with the OBZ-POSS thin film [108]. Reproduced with permission from Elsevier.

The TGA data indicated that the weight loss decreased after heating with temperature higher than 550 °C for both samples of OBZ-POSS and P-pa and the incorporation of POSS NPs into the PBZ matrix enhanced its thermal stability through the formation of a crosslinking network structure after ring opening of the BZ units on the inorganic silsesquioxane. In addition, this OBZ-POSS material displayed a low surface free energy, measured from water contact angles, after modification of the thin film with poly(4-vinylpyridine) (Figure 7).

**Figure 8:**
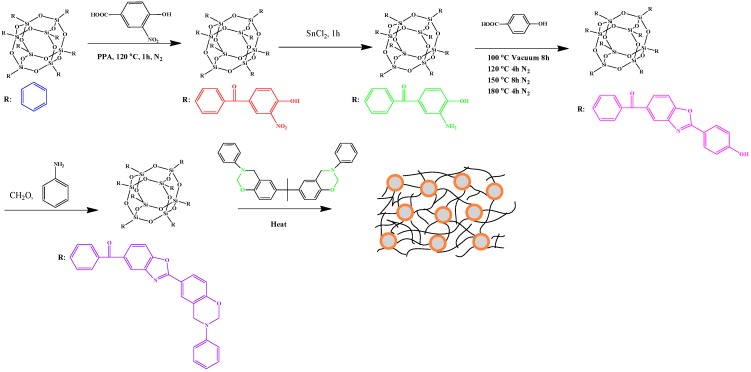
Synthesis of OPS-BZ and possible morphology of POSS/PBZ nanocomposites after thermal curing [109]. Reproduced with permission from American Chemical Society.

Han *et al.* [109] prepared a class of benzoxazole-modified [PhSiO_1.5_]_8_ BZ (OPS-BZ) monomers and blended them with BA-a to afford POSS/PBZ nanocomposites (Figure 8).

**Figure 9:**
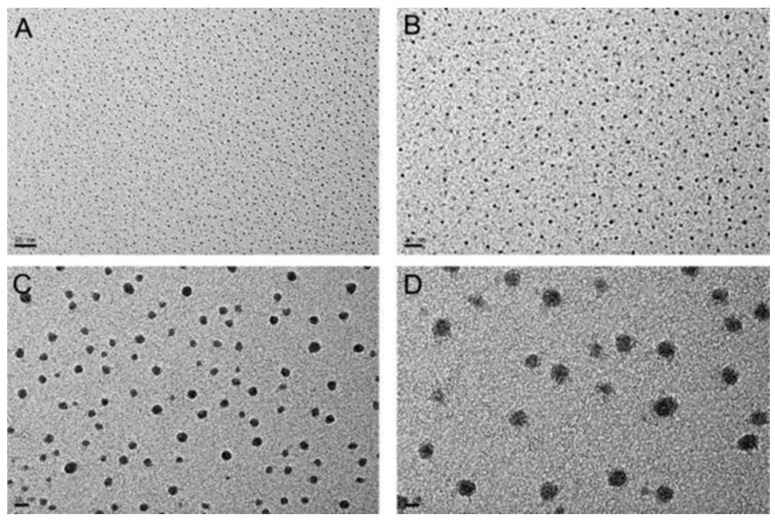
HR-TEM images for POSS/PBZ =30/70 nanocomposites (scale bars for **A**: 50 nm; **B**: 20 nm; **C**: 10 nm, and **D**: 5 nm) [109]. Reproduced with permission from American Chemical Society.

They used high-resolution transmission electron microscopy (HR-TEM) to study the microstructure of the POSS/PBZ (30/70 wt %) nanocomposite (Figure 9) [109]. The TEM images revealed highly dispersed POSS units (size: 3–10 nm) in the PBZ matrix, due to the rigid benzoxazole group around the OPS core minimizing the aggregation of the POSS NPs. Moreover, these OPS-BZ/PBZ nanocomposites possessed low dielectric constants and dielectric losses for frequencies in the range from 10 Hz to 1 MHz.

**Figure 10:**
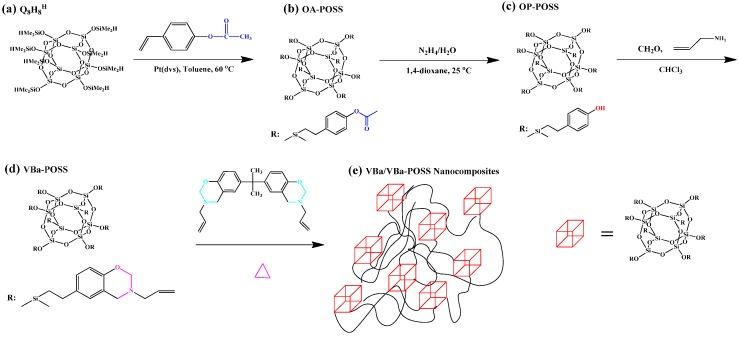
(**a**–**d**) The reaction and chemical structures for the preparation from Q_8_M_8_^H^ (**a**) to form OA-POSS (**b**), OP-POSS (**c**), and VBa-POSS (**d**); the possible morphology of VBa/VBa-POSS blends after the thermal curing (**e**) [110]. Reproduced with permission from John Wiley and Sons.

We also developed a new PBZ/POSS nanocomposite prepared from the reactions of a multifunctional vinyl-terminated POSS derivative (VBa-POSS) and the VBa BZ monomer at various compositions (Figure 10) [110]. These hybrid materials exhibited good thermal stability because the incorporation of the POSS units into the PBZ resins hindered the mobility.

**Figure 11:**
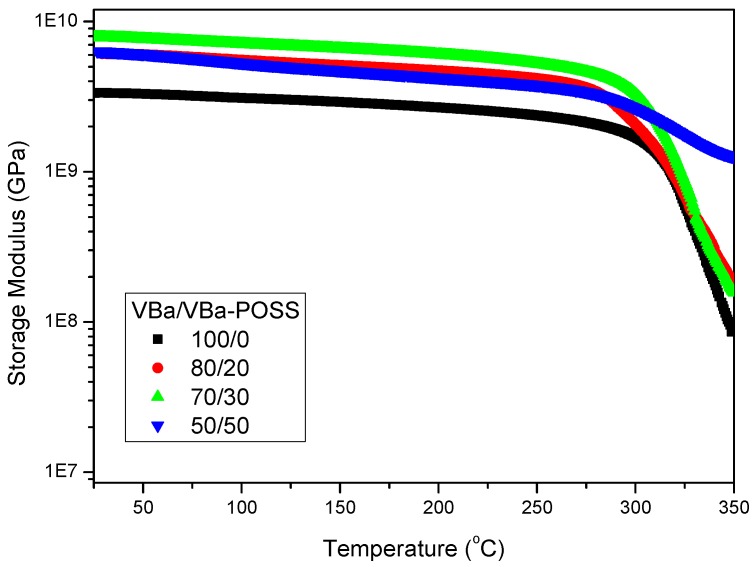
Storage moduli of VBa/VBa-POSS nanocomposites after thermal curing [110]. Reproduced with permission from John Wiley and Sons.

In addition, these poly(VBa/VBa-POSS) hybrid materials displayed enhanced mechanical and thermal properties after curing (Figure 11) because (i) the bulky POSS cores tend to stiffen the crosslinking network structure and (ii) the hydrogen-bonding interaction existed between the hydroxyl (OH) groups of the PBZ moieties and the siloxane units in the POSS cores after thermal polymerization. 

**Figure 12:**
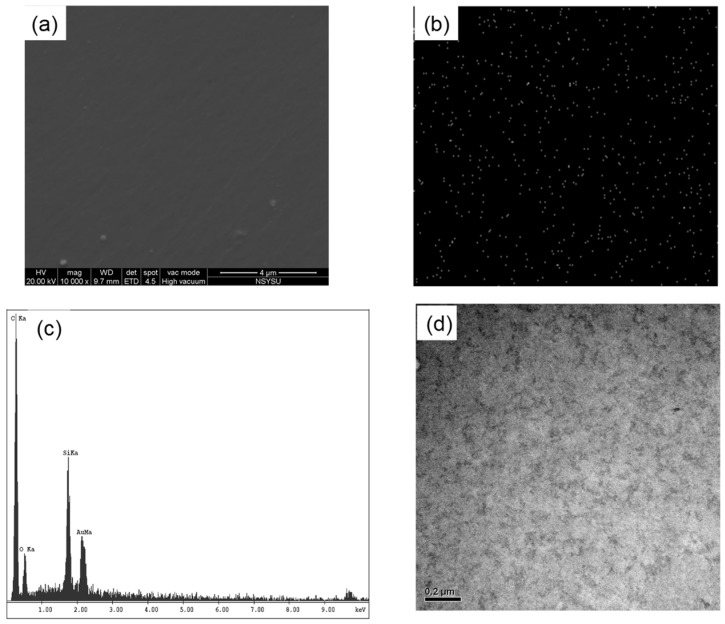
SEM image (**a**), Si-mapping image (**b**), EDX analysis (**c**), and TEM image of VBa/VBa-POSS = 70/30 after thermal curing (**d**) [110]. Reproduced with permission from John Wiley and Sons.

Moreover, scanning and transmission electron microscopy of cured VBa/VBa-POSS (70/30) revealed uniformly dispersed spherical POSS NPs throughout the PBz matrix, without any discernible phase separation (Figure 12a,b).

**Figure 13:**
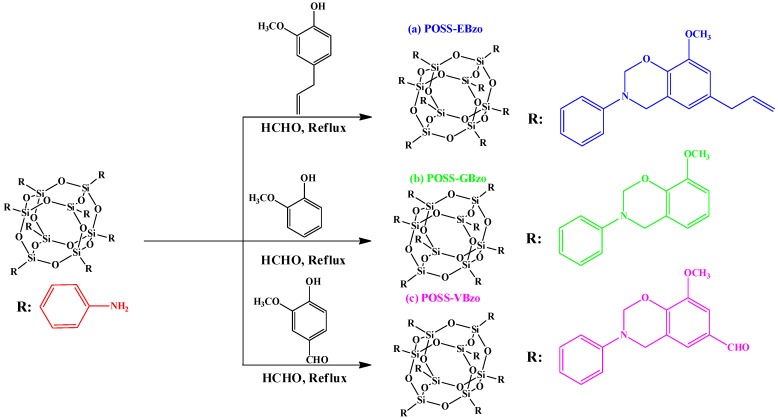
Synthesis of POSS-BZ monomers of POSS-EBzo (**a**), POSS-GBzo (**b**), and POSS-VBzo (**c**) [111]. Reproduced with permission from Royal Society of Chemistry.

Recently, Muthusamy *et al.* [111] synthesized PBZ-tethered polyhedral oligomeric silsesquioxane nanocomposites through condensation of eugenol (E), guaiacol (G), and vanillin (V) with POSS octamine and paraformaldehyde in DMSO at 130 °C for 5 h (Figure 13).

**Figure 14:**
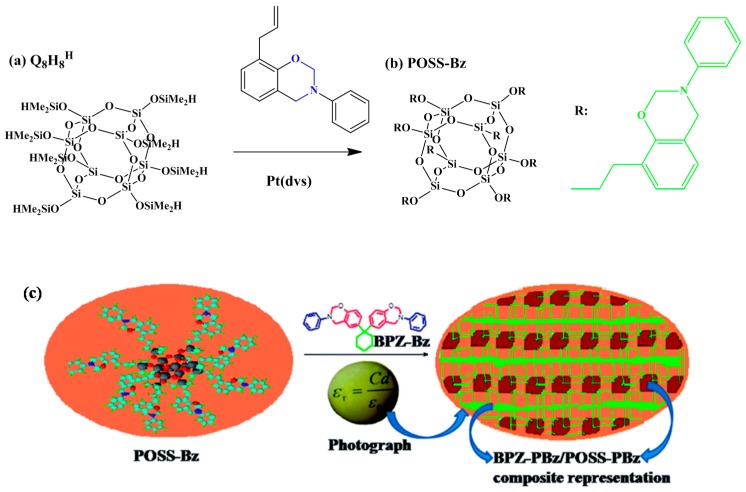
(**a**) Q_8_M_8_^H^; (**b**) synthesis of POSS-Bz; (**c**) the schematic representation of BPZ/POSS nanocomposite with the cross-linked lamellae structure [112]. Reproduced with permission from Royal Society of Chemistry.

SEM images of the POSS-EPbz, POSS-GPbz, and POSS-VPbz indicated that the POSS NPs remained well dispersed in this PBZ resin. In addition, AFM images of these hybrid materials revealed rough areas corresponding to the POSS domains and smooth areas corresponding to the PBZ regions. The dielectric constant and dielectric loss of such materials are strongly dependent on their chemical structures, porosities, and polarizabilities [111]. The values of dielectric constant of POSS-EPbz, POSS-GPbz, and POSS-VPbz hybrid materials were 1.98, 1.85 and 1.88, respectively. The relatively low dielectric values of these hybrid nanocomposite materials presumably arose from the uniformity of the dispersion of the POSS units throughout the matrix and from the low polarity of the POSS units themselves. Kumar *et al.* [112] observed a new class of lamellar structure of POSS/bisphenol Z (POSS/BPZ) nanocomposites during the ring opening polymerization of BZ (Figure 14). They found that this lamellar structure for the 30% POSS-PBZ nanocomposite had an ultralow value of *k* (1.7).

### 2.3. Other Functionalized POSS Derivatives in PBZ

**Figure 15:**
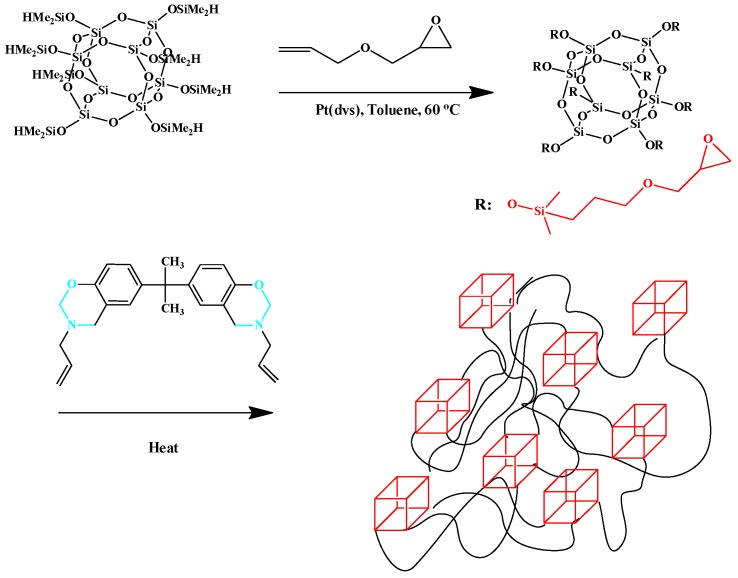
Preparation of OG-POSS and the subsequent reaction with VBa monomer to give PBZ/POSS nanocomposites [113]. Reproduced with permission from John Wiley and Sons.

The introduction POSS NPs into PBZ matrices can be performed not only with BZ-functionalization but also with other functionalized groups. For example, Zheng *et al.* and our own group incorporated octafunctionalized epoxy POSS into a BZ monomer to form PBZ/POSS nanocomposites (Figure 15) [113,114].

**Figure 16:**
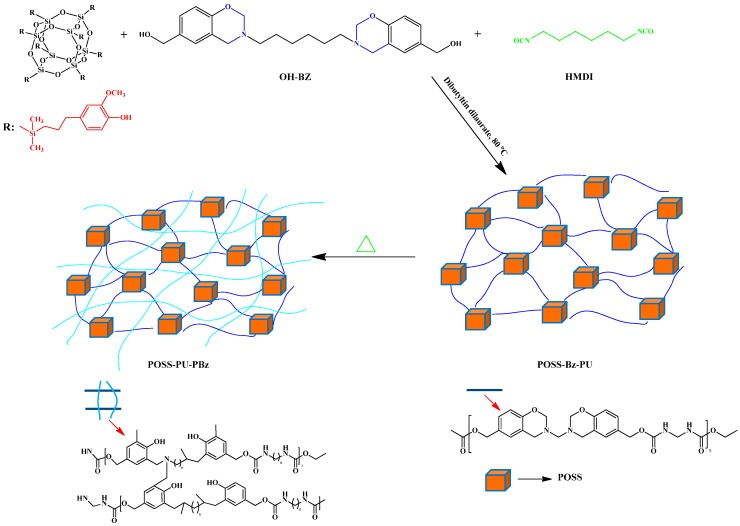
Preparation of POSS-PU-PBZ nanocomposites [116]. Reproduced with permission from Royal Society of Chemistry

Using thermal curing polymerization, Selvi *et al.* [115] prepared transparent and homogenous reinforced PBZ/EP/OG-POSS nanocomposites after reinforcing OG-POSS at various weight ratios into BA-a monomer and DGEBA epoxy (EP) matrices. They observed distinct dark spots (*ca.* 40 nm) representing well-dispersed POSS core units within the PBZ/DGEBA epoxy matrix after thermal polymerization, as well as improved resistance of these hybrid materials toward UV radiation, attributed to the presence of the inert silica layer on the composite surface. Alagar *et al.* prepared a low-*k* nanocomposite material through copolymerization of OH-BZ and OH-POSS with hexamethylene diisocyanate (HMDI) to afford POSS-BZ-PU, with subsequent thermal curing polymerization giving the POSS-PU-PBZ nanocomposite material (Figure 16) [114].

After reinforcing PU-PBZ with 30 or 40 wt% of POSS, the degradation temperature (*T*_d10_) and char yield of the nanocomposite (326.6 °C/33.9% and 350.2 °C/36.8%, respectively) were higher than those of the PU-PBZ (321.0 °C/9.8%) after thermal curing. In addition, the 30% POSS-PU-PBZ nanocomposite possessed a dielectric constant (1.94) lower than pure PU-PBZ because of the lack of agglomeration of the POSS NPs and the presence of the porous network structure, as revealed in the SEM and HRTEM images.

### 2.4. Hydrogen-Bonding Interactions from Heteronucleobase-Functionalized BZ and POSS

**Figure 17:**
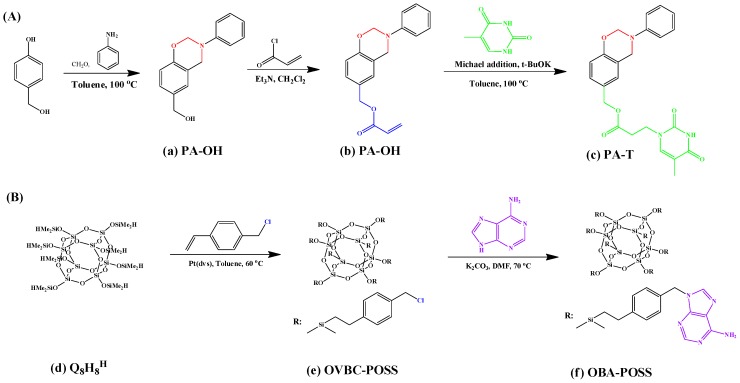
Syntheses of (**A**) PA-OH, PA-ac, and PA-T and (**B**) OBA-POSS [117]. Reproduced with permission from American Chemical Society.

**Figure 18:**
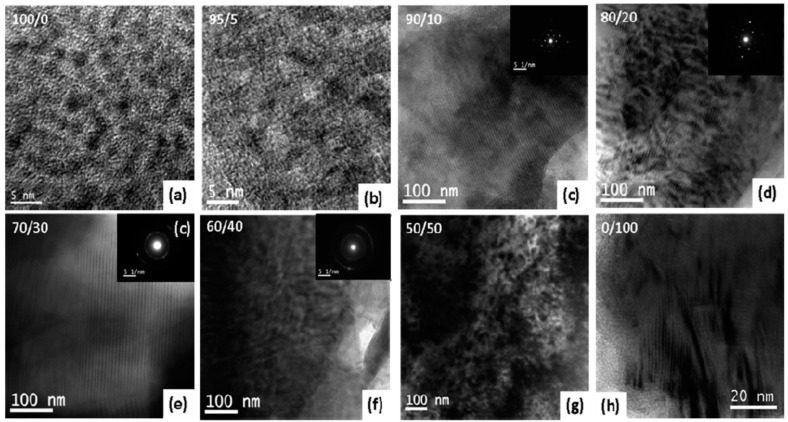
The TEM images and their corresponding SAED patterns of PA-T/OBA-POSS nanocomposites with different OBA-POSS contents of (**a**) 100/0, (**b**) 95/5, (**c**) 90/10, (**d**) 80/20, (**e**) 70/30, (**f**) 60/40, (**g**) 50/50, and (**h**) 0/100 [117]. Reproduced with permission from American Chemical Society.

**Figure 19:**
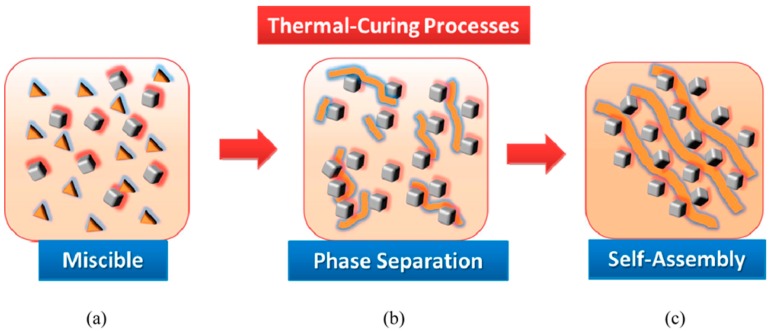
Possible three-step mechanism for self-assembly structure of PA-T/OBA-POSS hybrid complexes: (**a**) PA-T was miscible with OBA-POSS; (**b**) the OBA-POSS separated from the thermal cured PA-T through the reaction-induced microphase separation mechanism; (**c**) the self-aggregation of OBA-POSS was restricted by complementary A–T multiple hydrogen-bonding interaction and the self-assembly lamellae structure of the POSS units via the subsequent growth along (012) plane [117]. Reproduced with permission from American Chemical Society.

We have prepared octuply adenine (A)-functionalized POSS (OBA-POSS) NPs through hydrosilylation of A with the octakis(benzyl chloride) POSS (OVBC-POSS); this compound formed complementary multiple hydrogen bonding with thymine (T) groups of PA-T upon physical blending in THF solution (Figure 17) [49,117]. The second heating DSC scans of PA-T/OBA-POSS nanocomposites revealed two interesting phenomena:

(i) the glass transition temperatures (*T*_g_) of PA-T/OBA-POSS nanocomposites were higher than that of neat OBA-POSS and (ii) the value of *T*_g_ of the hybrid materials decreased with the increase of OBA-POSS contents, because of the aggregation of POSS units and the occurrence of macro-phase separation. In addition, TGA results revealed that upon incorporation 20 wt % of OBA-POSS, the thermal stability of PA-T was greater than those neat PA-T and OBA-POSS, presumably because of intermolecular hydrogen-bonding interaction and formation of network structure PA-T and inorganic POSS. The TEM image and selected area electron diffraction (SAED) pattern of pure PA-T and the PA-T/OBA-POSS nanocomposites (Figure 18) revealed that the former self-assembled into an ordered lamellae structure [117] whereas the latter formed the long range order structure within the PBZ matrix, as displayed in Figure 19.

**Figure 20:**
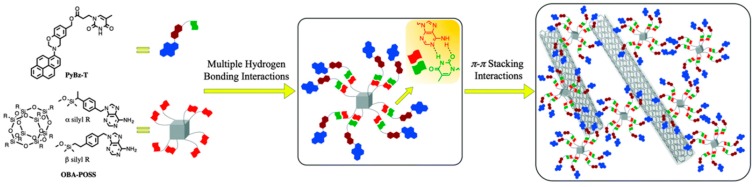
The cartoon representation of the Py-Bz-T/OBA-POSS/SWCNT ternary hybrid complexes formation [118]. Reproduced with permission from Royal Society of Chemistry.

**Figure 21:**
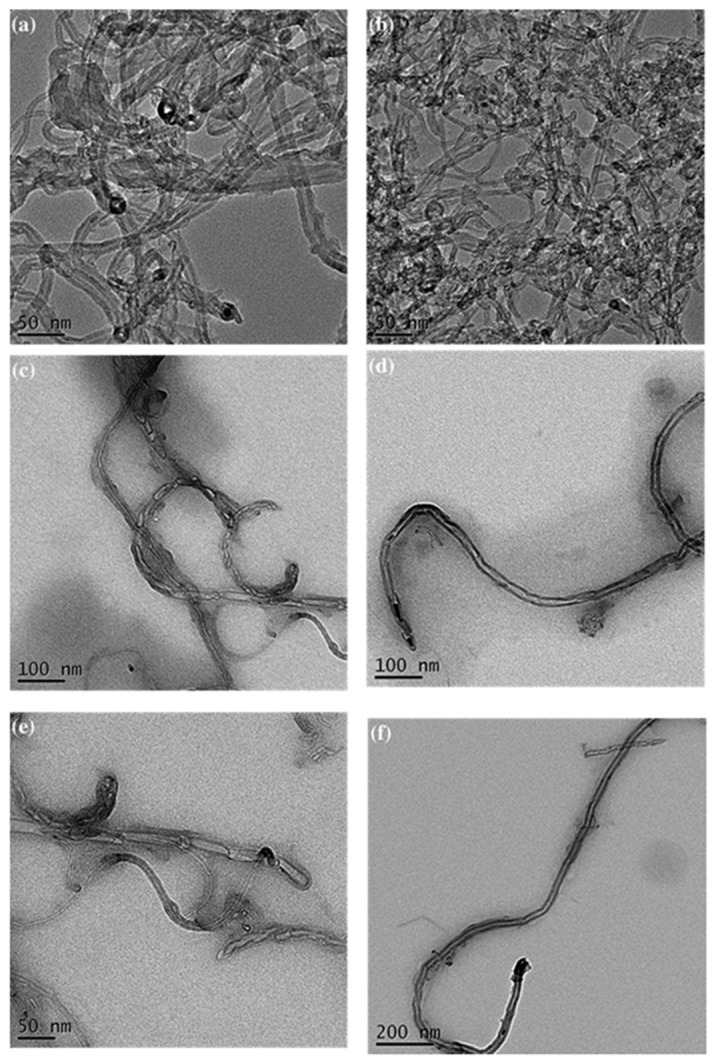
TEM images of pure SWCNT (**a,b**), the Py-Bz-T/OBA-POSS/SWCNT (1 wt%) (**c,d**), and the Py-Bz-T/OBA-POSS/SWCNT (3 wt %) hybrid complex (**e,f**) [118]. Reproduced with permission from Royal Society of Chemistry.

We have also prepared ternary nanocomposites of zero-dimensional POSS and one-dimensional single walled carbon nanotube (SWCNT) linked throughout T-functionalized PBZ matrix, stabilized via the multiple hydrogen bonding and the π–π stacking interaction (noncovalent supramolecular interactions) as presented in Figure 20 [118]. TEM images of these PBz/POSS/SWCNTs hybrid materials revealed (Figure 21) that the SWCNTs were well dispersed within the PBZ matrix.

## 3. Conclusions

Many different kinds of PBZ/POSS nanocomposites have been prepared displaying superior thermal stability, higher glass transition and degradation temperatures, and lower dielectric constants than those of pristine BZ monomers and PBZ polymers. POSS NPs have several attractive features that make them attractive alternatives to traditional fillers or clay, including well-defined structures, an absence of trace metals, highly uniform dispersion within polymer matrices, and good interfacial interactions with the polymer segments. The incorporation POSS NPs into PBZ matrix has been performed through both chemical crosslinking and physical blending. In this review, we have discussed several different classes of PBZ/POSS hybrid materials formed from mono- and multi-functionalized POSS through both covalent and noncovalent bonding. According to literature reviews, they reported that the incorporation POSS nanoparticles and DNA bases into polybenzoxazine could be enhanced the thermal and mechanical properties of PBZ. The preparation of such organic/inorganic hybrid materials containing POSS and PBZs remains one of the hottest topics in academic and industrial research because of their potential applications in, for example, drug delivery and microelectronic devices, and for their use as low-*k* materials (e.g., as insulators).
